# Completeness and generalizability of the Swedish MS register

**DOI:** 10.1007/s10654-025-01276-z

**Published:** 2025-07-24

**Authors:** Peter Alping, Thomas Frisell, Anna He, Jan Hillert, Katharina Fink, Kyla A. McKay

**Affiliations:** 1 Division of Clinical Epidemiology, Department of Medicine, Karolinska Institutet, Maria Aspmans gata 30A, 171 64, Solna, Stockholm, Sweden; 2 Department of Clinical Neuroscience, Karolinska Institutet, Stockholm, Sweden; 3 Department of Neuroinflammation, Queen Square Institute of Neurology, University College London, London, UK; 4 Department of Neurology, Karolinska University Hospital, Stockholm, Sweden; 5 Centre for Neurology, Academic Specialist Centre, Stockholm, Sweden; 6 Center for Molecular Medicine, Karolinska University Hospital, Stockholm, Sweden

**Keywords:** Multiple sclerosis, Register, Validation, Sweden

## Abstract

The Swedish Multiple Sclerosis (MS) Register is a nationwide clinical tool and research database. Its generalizability to the broader MS population has not been previously assessed. We aimed to quantify the register’s completeness and potential disparities between those who were included and those who were not. Using linked national registers, we identified persons with MS through December 2020 as those with ≥ 3 MS diagnoses in the National Patient Register. We assessed the MS register’s completeness and compared characteristics between included and non-included persons, using overlap weighting to account for age differences. Among 21,320 prevalent (2020) and 7,355 incident (2011–2020) MS cases, the completeness of the MS register was 85% and 87%, respectively, but varied by county (67–94% in 2020). Prevalent cases not included in the register were older (mean 62.4 vs. 52.3 years) and more likely to have no salaried income (55.3% vs. 30.5%); had fewer years of education (20.8% vs. 12.0% with ≤ 9 years); more often been hospitalized (41.5% vs. 32.2%); and collected more unique prescribed drugs (median 7 vs. 5 drugs per year). Incident cases were also older at first MS diagnosis (mean 50.3 vs. 39.2 years). All differences were attenuated by adjusting for age, although disparities remained. The Swedish MS Register demonstrates high completeness, but may not fully represent older persons with MS, those diagnosed later in life, and those of lower socioeconomic status. These limitations should be considered when interpreting findings based on the register’s data.

**Supplementary Information:**

The online version contains supplementary material available at 10.1007/s10654-025-01276-z.

## Introduction

The Swedish Multiple Sclerosis (MS) Register is a publicly funded nationwide quality-of-care register, officially launched in 2001 to extend the clinical documentation and prospectively collect data for improving medical care [1–3]. It is used by healthcare providers to monitor disease, by policymakers to make healthcare decisions, and by researchers to better understand MS at the population level. Data are recorded by physicians, nurses, and the patients themselves, and all counties and clinics offering specialist neurological care in Sweden are represented, with the goal that all MS patients in Sweden should be offered to be included [3].

Although participation is voluntary, the completeness of the MS register has been estimated to be over 80%.^3,4^ Previous validations of the register have assessed its data quality, completeness, and specificity [4–6]. However, no study has investigated differences in characteristics between those who are included in the MS register and those who are not, which is critical to understanding the generalizability of the register to the broader MS population.

Therefore, we aimed to quantify the completeness of the Swedish MS register compared with the National Patient Register and assess potential disparities between the persons with MS that were included in the MS register and those that were not.

## Methods

We linked the Swedish MS register to national health and demographic registers, using the Swedish personal identification number, which is unique to each person [7]. A person was included in the linkage if they had, at any time, at least one visit to inpatient or specialized outpatient care with a diagnosis that could indicate MS (International Classification of Diseases [ICD]-10: G049, G359, G378, G379, H469) or was registered in the Swedish MS register. From this data linkage, persons with MS were identified for inclusion in the study (see below). This study was approved by the Swedish Ethical Review Authority (reference: 2021–02384 and 2023-07906-02).

### Setting and data sources

The Swedish healthcare system provides universal access with tax-funded, capped out-of-pocket costs for both prescription drugs and inpatient/outpatient care. Persons with MS are primarily diagnosed and followed at public neurology clinics, where the Swedish MS register is used as a clinical tool to document and evaluate disease progression and treatment response. Registration in the MS register is not mandatory, but all specialist neurology clinics in Sweden are represented. Follow-up in the register ends at emigration or death, but the data remain and the person can be re-enrolled in the event of re-immigration. If a registered individual is later found to have been misdiagnosed, their information is removed from the register.

The National Patient Register includes all inpatient care since 1987 [8]. Since 2001, this register also covers both public and private specialized outpatient visits to a physician. Primary care visits are not included. Among the information recorded for each visit are the visit date and the main and auxiliary diagnosis codes (ICD-9/10). Data in the National Patient Register have been verified to be of good quality, with high validity and low underreporting in recent years, especially for more severe diseases such as MS [9].

The Total Population Register provided information on birth, death, emigration and immigration dates, as well as region of origin [10, 11]. Annualized earnings from paid work (salaried income) was provided by the Income and Taxation Register [12]. Finally, highest achieved education level was provided by the Longitudinal Integration Database for Health Insurance and Labor Market Studies (LISA) [13, 14].

### MS definition and study population

We identified persons diagnosed with MS until December 31st, 2020, as those with at least three MS diagnoses (ICD-9/10: 340/G35, main or auxiliary diagnosis) registered in the National Patient Register, on separate dates, considering both inpatient and specialized outpatient visits. This definition has previously been found to have a well-balanced sensitivity and specificity [6]. Data from all sources were available until April 1st, 2024, allowing for at least three years of look-ahead time to fulfil the criteria (see Figure S1). Persons were censored at first emigration or death.

We considered the *intended population* of the Swedish MS register (i.e. the population that the register intends to include) to be every person in Sweden diagnosed with MS and the *included population* to be every person registered with MS in the MS register. The intended population was approximated using data from the National Patient Register and the above MS diagnosis definition.

In the intended population, we further classified individuals as *prevalent* (annually on December 31st, from 2005 to 2020) and *incident* (in the period 2011–2020) MS cases (not mutually exclusive, see Figure S1). To be considered a prevalent case, a person must have been diagnosed with MS according to the above definition and been living in Sweden at the specified timepoint. To be considered an incident case, a person must have been diagnosed with MS according to the above definition, had their first-ever MS diagnostic code recorded as a primary diagnosis between 2011 and 2020, lived in Sweden at the time of their first MS diagnosis, and had at least 10 years of look-back time from the first MS diagnosis date (i.e. lived in Sweden continuously, to ensure no previous MS diagnoses during that time). From here on, the term “MS diagnosis” refers to the first recorded ICD code for MS in the National Patient Register.

### Completeness and positive-predictive value

We defined the MS register’s *completeness* as the proportion of the intended population that was also included in the MS register,

1 $$\text{Completeness}=\frac{\left|\text{Included}\:\cap\:\text{Intended}\right|}{\left|\text{Intended}\right|}$$

and the *positive-predictive value* (PPV) as the proportion of those included in the MS register that were also in the intended population.

2 $$\:\text{PPV}=\frac{\left|\text{Intended}\:\cap\:\text{Included}\right|}{\left|\text{Included}\right|}$$

The completeness and PPV of the MS register were estimated for the prevalent cases for each applicable year. The completeness was also stratified by county in Sweden for the year 2020, as healthcare in Sweden is organized at the county/regional level.

### Differences in characteristics

To assess the MS register’s generalizability, demographic and health characteristics in the intended population were compared between those included in the MS-register and those not. For the prevalent cases, the comparison was done at the time of the prevalence assessment and for the incident cases at the time of the first ever MS diagnosis. Assessed variables included: age, sex, country of birth (Nordic [Denmark, Finland, Iceland, Norway, Sweden, Faroe Islands, Greenland, Åland]/not Nordic), education level (highest achieved, categorized as ≤ 9 years [primary], 10–12 years [secondary], and ≥ 13 years [higher education]), civil status (unmarried, married, divorced, widowed), county in Sweden, no salaried income (last three years), yearly salaried income (mean over the last three years for those who had an income), any visits to inpatient care (last three years), yearly number of visits to specialized outpatient care (mean over the last three years), and yearly number of unique drugs collected from pharmacies (mean over the last three years). The differences between those included in the MS register and those who were not were quantified as standardized mean differences to get a comparable measure between the groups across variables.

To further assess the differences in characteristics independent of age at the time of assessment (prevalent) or at first MS diagnosis (incident), we used overlap weighting to create a pseudopopulation in which age was equally distributed between the two groups. Propensity scores were estimated from a logistic regression model as the predicted probability of being in the MS register, dependent on age at first MS diagnosis expressed as a third order polynomial (to allow for a more flexible relationship). Overlap weights were then assigned as the probability of having the counterfactual MS register status (i.e. $\text{propensity score}$ for those not in the MS register and $1-\text{propensity score}$ for those included in the MS register) [15].     

## Results

We identified 21,320 persons with prevalent MS (on December 31st 2020) and 7,355 persons with incident MS (between 2011 and 2020), in the National Patient Register (see Tables 1 and 2). Among these, 18,153 (85%) of prevalent cases and 6,416 (87%) of incident cases were also in the MS register.

**Table 1:**
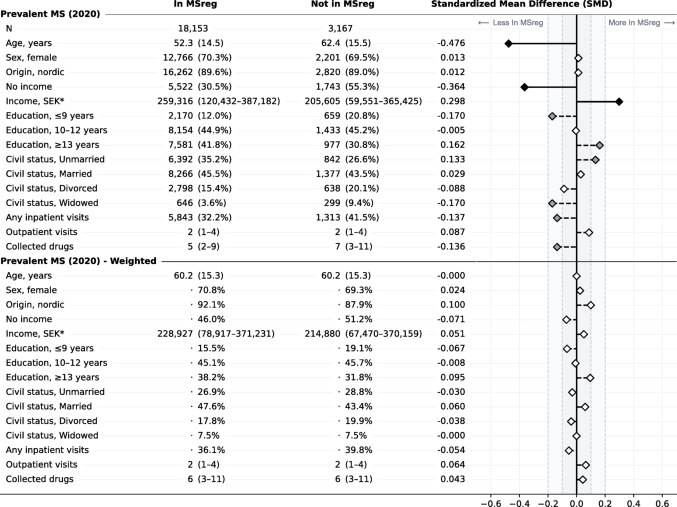
Population characteristics for the prevalent MS population on December 31st 2020

Stratified by if the persons were included in the MS register or not. Values are shown as number (percent), mean (standard deviation), or median (interquartile range). Differences between the two groups expressed as standardized mean differences (SMD). For the weighted population, the groups were made comparable by overlap weighting based on age at the time of assessment (December 31st 2020). *Income for those with any income

**Table 2:**
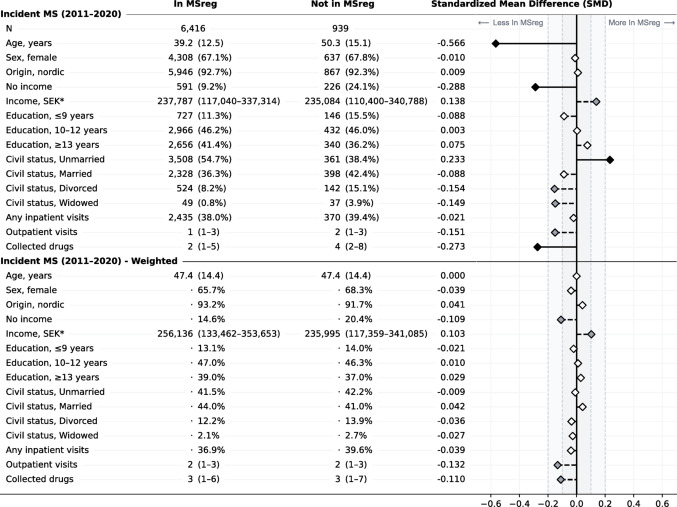
Population characteristics for the incident MS population (2011–2020) at the time of their first MS diagnosis

Stratified by if the persons were included in the MS register or not. Values are shown as number (percent), mean (standard deviation), or median (interquartile range). Differences between the two groups expressed as standardized mean differences (SMD). For the weighted population, the groups were made comparable by overlap weighting based on age at the time of first MS diagnosis. *Income for those with any income

### Completeness and positive predictive value

**Fig. 1:**
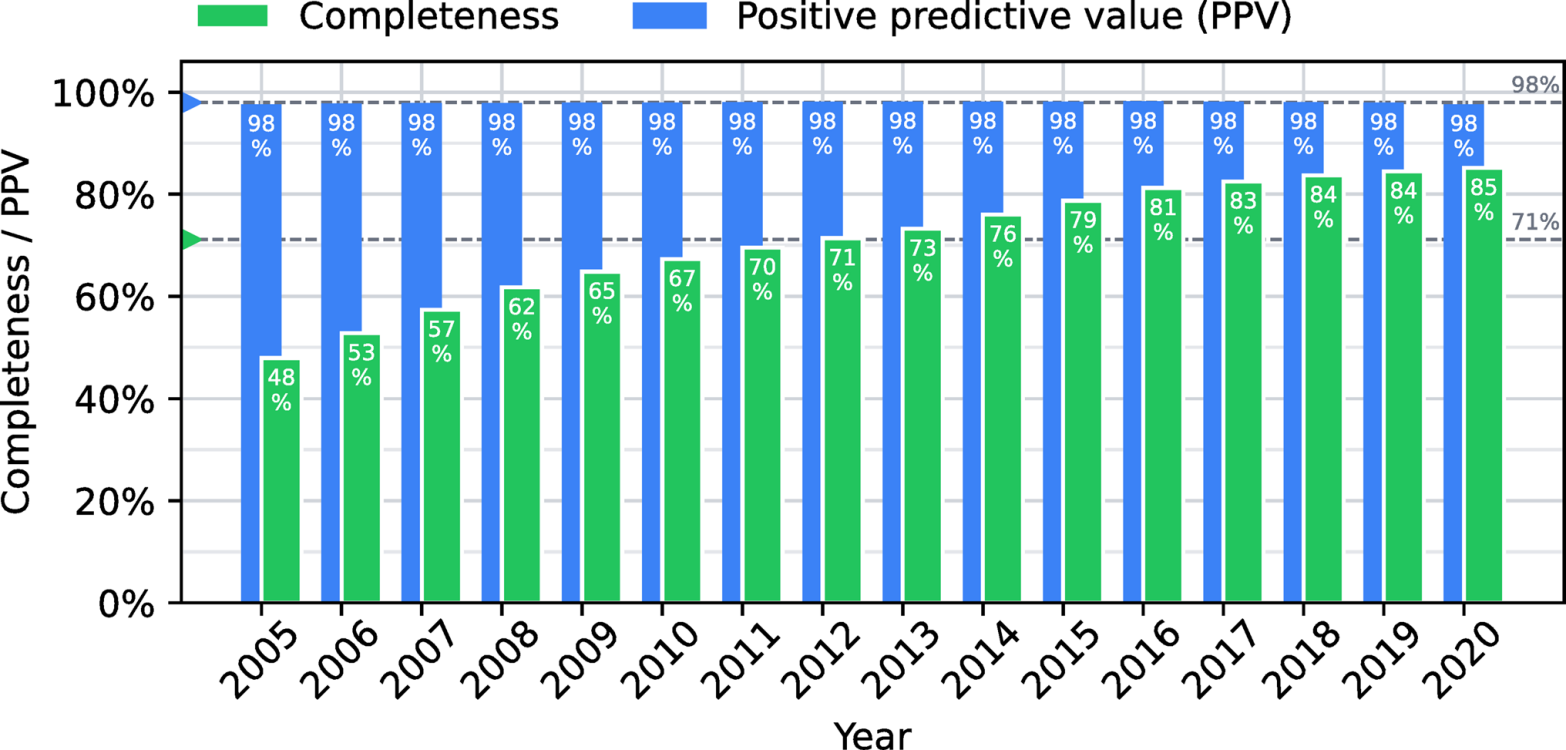
Completeness and positive predictive value (PPV) for prevalent MS by year. Mean values over the entire time period are shown as dashed lines

**Fig. 2:**
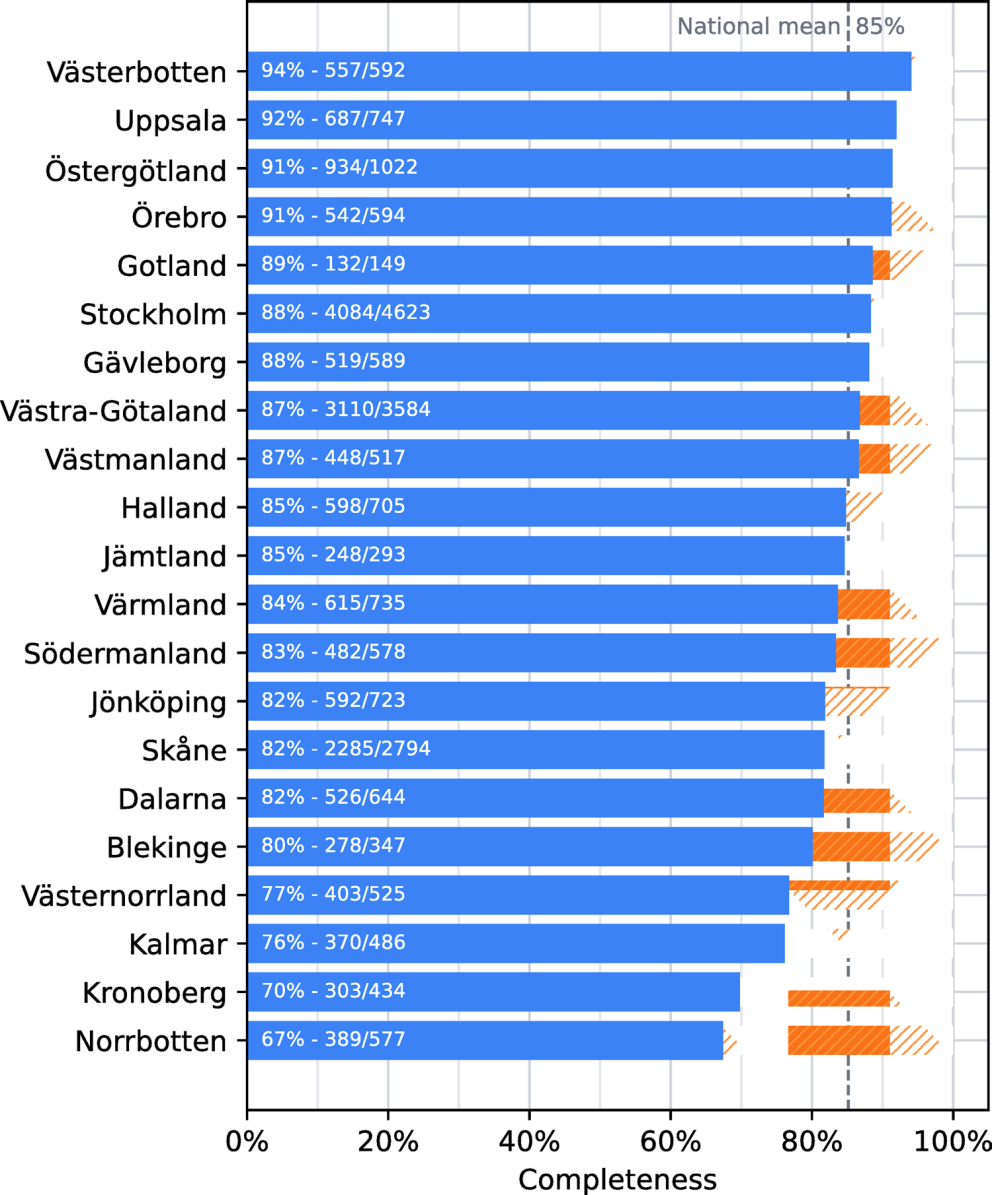
Completeness by county. The numbers on the bars show the completeness, the number of persons in the MS register, and the number of persons in the included population for each county. The national mean is shown as a dashed line

Between 2005 and 2020, mean completeness of the MS register for prevalent MS was 71%, with significant improvement over time, leading to 85% completeness in 2020 (Fig. 1). Completeness also differed by county, with a range of 67–94% (Norrbotten–Västerbotten) in 2020 (Fig. 2). The mean positive predictive value of the register (relative our approximation of the intended population) was 98% and was stable over time (Fig. 1). The time between first MS diagnosis and registration in the MS register decreased over time from a median of 4 months in 2011 to less than two weeks in 2020.

### Differences in characteristics

There were significant demographic differences between those included in the MS register and those who were not, both for the prevalent and incident cases (see Tables 1 and 2). Among prevalent cases, those not in the MS register were older (mean 62.4 vs. 52.3 years) and more likely to not have any salaried income (55.3% vs. 30.5%) or a lower salaried income if employed (median 205,605 vs. 259,316 SEK per year), have less education (20.8% vs. 12.0% with ≤ 9 years of education), be widowed (9.4% vs. 3.6%), have been hospitalized (41.5% vs. 32.2%), and have collected more prescribed drugs (median 7 vs. 5 different drugs per year), compared with those included in the register, at the time of prevalence assessment (December 31st, 2020). Among incident cases, those not in the MS register were older at first recorded MS diagnosis code (mean 50.3 vs. 39.2 years) and more likely to not have any salaried income (24.1% vs. 9.2%) or a lower salaried income if they had one (median 235,084 vs. 237,787 SEK per year), be divorced (15.1% vs. 8.2%) or widowed (3.9% vs. 0.8%), have more visits to specialized outpatient care (median 2 vs. 1 visits per year), and have collected more unique prescribed drugs (median 4 vs. 2 different drugs per year), compared with those included in the register, at the time of first MS diagnosis.

In the weighted pseudopopulations of prevalent and incident cases, where comparisons were independent of age at assessment and age at MS diagnosis, respectively, all previous differences were attenuated (see Tables 1 and 2). However, for the incident cases, minor differences remained between those not included in MS register and those who were, for salaried income (20.4% vs. 14.6% with no income and median 235,995 vs. 256,136 SEK for those with an income), outpatient visits (although with same median of 2), and unique collected drugs (although with same median of 3).

Additionally, there were differences in which county of Sweden a person was registered in at the time of prevalence assessment. A person with prevalent MS at December 31st, 2020, was more likely to be registered in the MS register if they were from Västerbotten, Uppsala, or Östergötland, and less likely if they were from Norrbotten, Kronoberg, or Kalmar (see Fig. 2).

## Discussion

In this first validation of the Swedish MS register assessing the generalizability to the general Swedish MS population, we found that completeness was high for recent years and PPV was high overall, but that there were differences between persons with MS that were included in the MS register and those that were not. Generally, individuals not in the MS register were older, had less income, and exhibited higher healthcare utilization than those included in the register.

The high completeness and PPV of the MS register are in line with previous reports. The Swedish Board of Health and Welfare (Socialstyrelsen) found, in a 2019 report of the MS register, a completeness of 80%, with large differences between counties (63–91%).^4^ A study in the Swedish county of Värmland estimated the completeness of the MS register between 2001 and 2013 to be 71% and found that every person in the MS register that underwent chart review (10% of total population) had a correct MS diagnosis [6]. Finally, the MS register’s own yearly audits, based on clinical chart review, have found no erroneous MS diagnoses in the register [3].

The completeness of the MS register has increased over time, reflecting the successful work of the neurologists and nurses to improve the uptake and use of the register. Nevertheless, our results show that the MS register is not used equally across the country. Interestingly, the top and bottom performers (Västerbotten and Norrbotten, respectively) are geographical neighbours, indicating that geographical location is, perhaps, less important than available healthcare resources in the county and local guidelines, experiences, and personnel at the specific treatment centres. This can, in part, be explained by Västerbotten’s and Norrbotten’s similarly sized, but very differently distributed populations. Västerbotten has a more centralized, urban population and hosts a university hospital that provides specialist care to the four northernmost counties in Sweden, including Norrbotten. Persons with MS in Västerbotten are therefore likely to have easier access to neurologists that use the MS register, compared with those living in Norrbotten.

The large difference in mean age for the prevalent MS cases indicates that the Swedish MS register is less generalizable to the older MS population. This could represent a tendency among physicians and nurses to prioritize inclusion of younger persons with MS, or could reflect the lower rates of MS register use in the past, when older patients were more likely to have first been diagnosed. Related, but with distinct implications, is the difference in age at first MS diagnosis among the incident MS cases, which signifies that persons presenting with MS later in life were less likely to be included in the MS register. It is possible that this group is composed of a higher proportion of persons with primary progressive MS (PPMS), which typically has a later onset compared with the more common relapsing-remitting MS (RRMS). During the study period, there were no approved disease-modifying therapies for PPMS; therefore, it is conceivable that these persons would not be included in the MS register, given that one of its primary clinical utilities is to track therapy use over time. However, PPMS is also more common in men than RRMS and we could not see any difference in the sex distributions between the two groups. Older adults with MS tend to exhibit greater physical disability, which increases their likelihood of requiring long-term home care, which could also lead to underuse of the register, as they do not visit neurology clinics. An alternative explanation is that those not in the MS register include a higher proportion of persons with “benign” MS, for which treatment and specialist neurological care were not sought, or that they were diagnosed prior to both the MS register and the availability of effective treatment, resulting in limited follow-up. Since we have no data on the clinical course for individuals not included in the MS register, extensive chart review would be necessary to test these competing hypotheses.

The other discrepancies between the groups were largely explained by the difference in age at assessment or first MS diagnosis (for the prevalent and incident cases, respectively), as reflected by the diminished differences between the groups in the weighted pseudopopulations. The attenuated, but remaining differences included salaried income, outpatient visit rates, and number of unique collected drugs before MS diagnosis. In short, these indicated that those not in the MS register earn less and bear a higher morbidity burden than those included in the register, in the period immediately before their MS diagnosis. This suggests that sociodemographic features may contribute to a person’s opportunity or decision to enrol in the MS register, which is important as effective interventions targeting social determinants of health could have substantial effects on MS outcomes [16]. To address these differences, the MS register should continue its work to reach out to neurological clinics and individual neurologists, and integrate the register into the existing clinical chart systems for easier access and registration. The MS register could work together with patient organizations to inform persons with MS about the benefits of taking part in the register, both for the individual and for the future of MS research.

## Strengths and weaknesses

The main limitations of this study were the lack of primary care data and remaining uncertainties around the MS definition and date of the first MS diagnosis (for the incident MS cases). The lack of primary care data is likely to primarily affect the prevalent MS cases, as we lose out on opportunities for when an MS diagnosis could have been registered, possibly leading to an underestimation of the Swedish MS population. However, as all MS patients in Sweden should, according to guidelines, be first diagnosed and then followed by a neurologist, the majority of MS patients will likely be captured by the National Patient Register given enough time, and especially around the time of the first MS diagnosis (lessening the impact of this on incident MS). Our MS definition requiring at least 3 MS diagnoses at separate dates has previously been validated against clinical charts and found to strike a good balance between sensitivity and specificity [6]. However, as our MS definition is unlikely to perfectly approximate the MS register’s intended population, there is a risk of underestimating both the completeness and PPV of the register. Given that previous reports have found no erroneous MS diagnoses in the MS register, the register’s true PPV is likely higher than reported in this study. Although the possibility of MS misdiagnosis remains, Sweden now maintains dedicated registers for neuromyelitis optica spectrum disorder (NMOSD) and myelin oligodendrocyte glycoprotein antibody disease (MOGAD), two diseases that mimic MS. Individuals found to be incorrectly registered as having MS are removed from the MS register and, if appropriate, added to the relevant disease-specific register. The validity of the date of the first MS diagnosis is crucial for the validity of the comparison of characteristics among the incident MS cases. To avoid erroneously including prevalent MS cases in this population, we chose a conservative set of inclusion criteria, requiring a long run-in period with full data coverage. This meant that for a person with prevalent MS to be misclassified as an incident case within the study period, the person would have had to live in Sweden for at least 10 years without any contact with specialized outpatient or inpatient care (where an MS diagnosis could have been recorded) during that time. It is therefore unlikely that the differences in characteristics in the incident MS population, between those included in the MS register and those that were not, could be explained by misclassification of the date of first MS diagnosis.

The main strengths of this study are the high-quality data of the National Patient Register, the strict definition of the incident MS population, and the long study period. The comprehensive, validated, nationwide data from the National Patient Register make it possible to approximate the Swedish MS population with a high degree of certainty. This together with the stringent definition of the incident MS population, requiring at least 10 years of look-back time and a first MS diagnosis as a main diagnosis, minimizes misclassification and provides high confidence in the temporal accuracy of the MS diagnoses. The long study period allows for robust assessments over time, providing insights into the register’s improving completeness.

## Conclusions

This is the first validation of the Swedish MS register assessing the generalizability to the broader MS population. We found that the register had overall high completeness (85% in 2020) and excellent PPV (98%), but was less complete for older persons with MS, persons with MS diagnosed later in life, and those of lower socioeconomic status. Despite the overall high completeness, generalizability needs to be considered in studies of these groups using the MS register data.

## Electronic supplementary material

Below is the link to the electronic supplementary material.

Supplementary Material 1
