# Supplementary material for: Completeness and generalizability of the Swedish MS register
Source: Eur J Epidemiol. 2025 Jul 24;40(8):905–13. doi: 10.1007/s10654-025-01276-z (PMC12374865; doi:10.1007/s10654-025-01276-z)
Supplement: Supplementary file 1 — Supplementary Material 1 [file 10654_2025_1276_MOESM1_ESM.docx]

# Supplementary Material

| 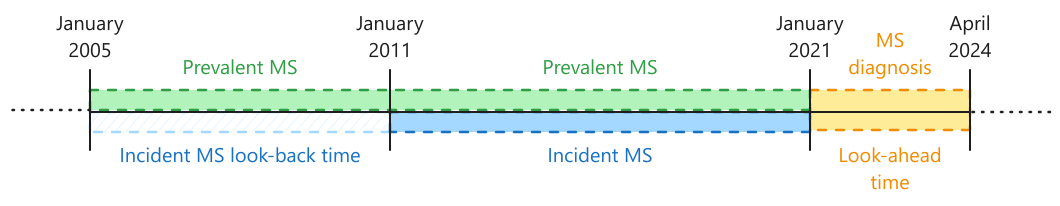  Figure S1: **Timeline for the definitions of MS diagnosis, prevalent MS, and incident MS.** |
| --- |
